# Hydrogen Peroxide Induced Toxicity Is Reversed by the Macrocyclic IRAP-Inhibitor HA08 in Primary Hippocampal Cell Cultures

**DOI:** 10.3390/cimb44100340

**Published:** 2022-10-18

**Authors:** Frida Stam, Sara Florén Lind, Anja Schroff, Sofia Zelleroth, Erik Nylander, Johan Gising, Alfhild Grönbladh, Mats Larhed, Mathias Hallberg

**Affiliations:** 1 The Beijer Laboratory, Department of Pharmaceutical Biosciences, Uppsala University, SE-75124 Uppsala, Sweden; 2 The Beijer Laboratory, Science for Life Laboratory, Department of Medicinal Chemistry, Uppsala University, SE-75124 Uppsala, Sweden

**Keywords:** primary cell cultures, Angiotensin IV, insulin-regulated aminopeptidase (IRAP), hippocampus

## Abstract

Angiotensin IV (Ang IV), a metabolite of Angiotensin II, is a bioactive hexapeptide that inhibits the insulin-regulated aminopeptidase (IRAP). This transmembrane zinc metallopeptidase with many biological functions has in recent years emerged as a new pharmacological target. IRAP is expressed in a variety of tissues and can be found in high density in the hippocampus and neocortex, brain regions associated with cognition. Ang IV is known to improve memory tasks in experimental animals. One of the most potent IRAP inhibitors known today is the macrocyclic compound HA08 that is significantly more stable than the endogenous Ang IV. HA08 combines structural elements from Ang IV and the physiological substrates oxytocin and vasopressin, and binds to the catalytic site of IRAP. In the present study we evaluate whether HA08 can restore cell viability in rat primary cells submitted to hydrogen peroxide damage. After damaging the cells with hydrogen peroxide and subsequently treating them with HA08, the conceivable restoring effects of the IRAP inhibitor were assessed. The cellular viability was determined by measuring mitochondrial activity and lactate dehydrogenase (LDH) release. The mitochondrial activity was significantly higher in primary hippocampal cells, whereas the amount of LDH was unaffected. We conclude that the cell viability can be restored in this cell type by blocking IRAP with the potent macrocyclic inhibitor HA08, although the mechanism by which HA08 exerts its effects remains unclear.

## 1. Introduction

**Figure 1:**
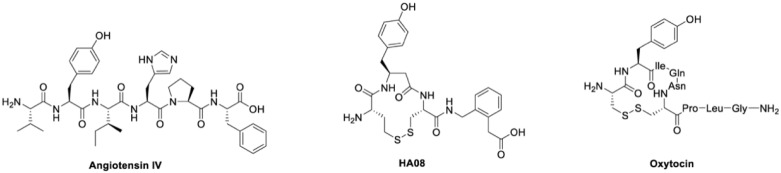
Chemical structures. The chemical structures of the endogenous IRAP inhibitor and cognitive enhancer angiotensin IV, the potent synthetic IRAP inhibitor HA08 and the IRAP substrate oxytocin [12].

Insulin-regulated aminopeptidase (IRAP; oxytocinase; placental leucine aminopeptidase; EC 3.4.11.3) is a member of the M1 family of aminopeptidases and participates in several important physiological processes [1,2]. IRAP is expressed in a variety of tissues, and a high density is reported in hippocampus and neocortex, brain regions associated with cognition [2,3]. The aminopeptidase degrades oxytocin, but also vasopressin and several other presumed in vivo substrates [4,5,6]. Furthermore, it was reported that translocation of glucose transporter type 4 (GLUT4) to the plasma membrane upon insulin stimulation is mediated by IRAP, and that IRAP is engaged in the processing of peptides for presentation onto MHC class I molecules [7,8,9]. IRAP plays important roles in antigen cross-presentation [10] and T-cell receptor signaling [11], but regarding drug development programs, most efforts have so far focused on its role in cognition [12]. The aminopeptidase activity of IRAP is competitively inhibited by the hexapeptide Angiotensin IV (Ang IV), a degradation product from the octapeptide Angiotensin II (Ang II) [13]. Ang IV demonstrates a very different pharmacological profile than the parent compound Ang II [14], the latter with a pronounced hypertensive effect. Ang IV and some structurally related analogues have been shown to improve performance in a number of memory tasks when injected into the brains of rats, as first demonstrated by Braszko in 1988 [15,16,17,18,19,20,21]. These observations stimulated an interest in developing improved IRAP inhibitors, more potent than the short-lived endogenous Ang IV, as a new class of cognitive enhancers and as potential therapeutics for treating a variety of cognitive disorders such as Alzheimer’s disease [22,23,24]. A large series of IRAP inhibitors have been reported as well as support for the hypothesis that these types of compounds could serve as suitable starting points for drug discovery projects aimed at developing cognitive enhancers useful in clinic [9,22,25,26,27,28,29,30,31,32]. A characteristic feature of IRAP is the ability to bind to cyclic substrates [33]. The macrocyclic compound HA08, a cyclic analogue of the linear Ang IV, designed to mimic the N-terminus of oxytocin and vasopressin, is one of the most potent IRAP inhibitors known today (Figure 1) [12,34]. The compound alters dendritic spine density in rat hippocampal primary cultures [30] and is significantly more stable than the endogenous IRAP inhibitor Ang IV. Furthermore, the crystal structure of IRAP with HA08 that combines structural elements from Ang IV and the physiological cyclic substrates oxytocin and vasopressin confirms that HA08 binds in the catalytic site of IRAP [35]. One important characteristic of HA08 is its high 2000-fold selectivity for IRAP over AP-N, IRAP; 3.3 nM versus > 7000 nM [34] and high selectivity versus the homologous enzymes ER aminopeptidase 1 (ERAP 1) and ER aminopeptidase 2 (ERAP 2) [36]. HA08 has attracted attention as a potential lead structure for more drug-like cognitive enhancers acting via augmenting synaptic plasticity [25,26,29,30,34].

It has been demonstrated that Ang IV [15] and other IRAP inhibitors have the ability to improve memory performance in several types of animal models [2,9,16,17,18,19,37,38] and that Ang IV elicits protective effects against induced ischemia [39,40]. We previously demonstrated that the potent IRAP inhibitor and macrocyclic Ang IV analogue HA08 increases dendritic spine density in primary hippocampal cell cultures, indicating that a positive cognitive effect could be expected in vivo [30]. However, less is known about the protective or restorative effects of the compound. Herein, we report the data from an investigation of the ability of HA08 to restore cell viability of damaged rat primary hippocampal and cortical cells.

## 2. Materials and Methods

In order to evaluate whether HA08 could restore rat primary cells subjected to hydrogen peroxide damage, the cellular viability was determined. The viability was assessed by measuring the mitochondrial activity using tetrazolium bromide salt (MTT). Furthermore, the cytotoxicity was assessed by measuring the release of lactate dehydrogenase (LDH). The distribution of cell types as well as the expression of IRAP in mixed primary cell cultures were visualized using immunocytochemistry (ICC).

### 2.1. Primary Cortical and Hippocampal Cell Cultures

All animal experiments performed in this study were approved by the local animal ethics committee in Uppsala (Dnr: 5.8.18-18550/2018). Tissues from the frontal part of cerebral cortex and hippocampus were harvested from embryonic day 17 Sprague Dawley (Charles River, Calco, Italy) rat foetuses. The tissue was enzymatically digested using trypsin, and a homogenous cell suspension of either cell type was acquired using DNase and mechanical dissociation by pipetting. The final cell suspension was dissolved in Gibcos neurobasal plus media (NBM; Thermo Fisher Scientific, Waltham, MA, USA) supplemented with 0.25% glutaMAX^TM^ (Thermo Fisher Scientific), 1% penicillin/streptomycin (Thermo Fisher Scientific) and 4% Gibcos B27 plus (Thermo Fisher Scientific). The cells were seeded on 96-well plates precoated with 50 µg/mL poly-D-lysine (Sigma-Aldrich, St. Louis, MO, USA) and kept in an incubator at 37 °C and 5% (*v/v*) CO_2_. After 3 days in vitro (DIV), a full media change was performed and thereafter media were changed twice per week until treatment was started.

### 2.2. Immunocytochemistry

Immunocytochemistry (ICC) was used to determine the number of cells positive for the neuronal marker microtubule-associated protein 2 (MAP2) and astrocytic marker glial fibrillary acidic protein (GFAP), to characterize the rat primary cultures. IRAP was also targeted in a separate ICC to confirm the expression of the receptor. The cell density for all plates used for ICC was 5 × 10^4^ cells per well.

The cells were fixated with 4% PFA on 8 DIV and then permeabilized with Triton X-100 (MAP2, GFAP) or Tween 20 (IRAP). The cells were incubated in 10% Normal donkey serum for 1 h RT to block unspecific binding. The antibodies mouse anti-MAP2 (Merck Millipore, Burlington, MA, USA) and rat anti-GFAP (Invitrogen, Waltham, MA, USA) were added in concentration 1:500 for 1 h in RT. The antibody rabbit anti-IRAP (Cell Signaling Technology, Danvers, MA, USA) was also incubated for 1 h in RT, but in concentration 1:250. An appropriate secondary antibody (Alexa 488 anti-mouse, Alexa 568 anti-rat and Alexa 568 anti-rabbit; Invitrogen) was added in concentration 1:500 for 1 h RT and kept away from light. The cell nuclei were stained with 2.5:500 4′,6-diamidino-2-phenylindole (DAPI; Sigma-Aldrich) and incubated for 40 min in RT and kept away from light.

### 2.3. Image Analysis

Images were visualized and acquired using ImageXpress (Molecular Devices, San Jose, CA, USA) mounted with a 20× objective. The acquired images were analysed using an automated ImageJ macro developed by the authors to estimate the amount of MAP2 positive cells (neurons) and GFAP positive cells (astrocytes). Briefly, a region of interest (ROI) of each stained cell nucleus was acquired using the DAPI channel. These ROIs were overlaid on either the MAP2 channel or GFAP channel and intensity measurement was performed. ROIs positive for MAP2 staining were considered neurons, while ROIs positive for GFAP staining were considered astrocytes. ROIs not positive for either MAP2 or GFAP staining were considered other cells, most likely other types of glial cells.

### 2.4. Mitochondrial Activity (MTT Assay)

The mitochondrial activity of the cells was measured using tetrazolium bromide salt (MTT; Sigma-Aldrich). MTT was added to the cells at a concentration of 0.8 mg/mL and then incubated for 30 min in 37 °C kept away from light. The cells were then lysed with 100% DMSO and incubated in the dark at RT for 10–15 min before they were measured in a plate reader (FLUOstar Omega, Ortenberg, Germany). Active mitochondria metabolise MTT to a purple formazan product that was measured for absorbance at 570 nm. The amount of metabolised MTT corresponds to the amount of colour developed in the well and can be interpreted as the level of mitochondrial activity. All plates analysed using MTT had a cell density of 1 × 10^5^ cells per well.

### 2.5. Membrane Integrity (LDH Assay)

The amount of lactate dehydrogenase (LDH) was measured in the cell media using a cytotoxicity detection kit (Sigma-Aldrich). This reaction mix was added to 50 µL of cell media and then incubated for 30 min in RT kept away from light before measurement in a plate reader (FLUOstar Omega). Triton X-100 was used as a control for maximum cytotoxicity. When the reaction mix interacts with LDH, a red formazan product is produced, which was measured for absorbance at 492 nm. The amount of LDH corresponds to the amount of colour developed in the well and can be interpreted as the level of membrane integrity. All plates analysed for LDH release had a cell density of 1 × 10^5^ cells per well.

### 2.6. Substance HA08

HA08 is a macrocyclic IRAP inhibitor. The synthesis has previously been reported [30,34]. HA08 was dissolved in 100% DMSO to a concentration of 1 × 10^−2^ M, aliquoted and stored in −20 °C.

### 2.7. Determining LD_50_ of Hydrogen Peroxide

To determine the appropriate concentration to induce cell toxicity with hydrogen peroxide, a dose-response study was performed. Primary cortical cells were treated for 24 h with hydrogen peroxide diluted with MQH_2_O in concentrations 5 × 10^−6^, 8 × 10^−6^, 2 × 10^−5^, 3 × 10^−5^, 5 × 10^−5^, 8 × 10^−5^, 2 × 10^−4^ and 3 × 10^−4^ M. The MTT and LDH assays were performed to determine the LD_50_ value.

### 2.8. Induced Cell Toxicity with Hydrogen Peroxide

On 8 DIV, a full media change was performed by removing the NBM and adding minimum essential media (MEM; Thermo Fisher Scientific) containing no additional supplements. MEM was used to avoid interactions with the hydrogen peroxide. The hydrogen peroxide was diluted with MQH_2_O to a final concentration of 1 × 10^−4^ M. The control cells were treated with MQH_2_O only. The cells were incubated with hydrogen peroxide for 24 h at 37 °C and 5% (*v/v*) CO_2_. After 24 h, 50 µL of the cell media from each well was transferred to a new plate to be analysed for LDH release.

### 2.9. Treatment of Damaged Cells with IRAP Inhibitor HA08

After 24 h of hydrogen peroxide treatment, the media were removed and fresh MEM was added to the cells on 9 DIV. HA08 was added to the cells at the final concentrations 1 × 10^−5^, 1 × 10^−6^, 1 × 10^−7^, 1 × 10^−8^ and 1 × 10^−9^ M in 0.1% DMSO. The control cells were treated with 0.1% DMSO. The cells were incubated with HA08 for 24 h at 37 °C and 5% (*v/v*) CO_2_. After 24 h, 50 µL of the cell media from each well was transferred to another plate to be analysed for LDH release and the cells were analysed for mitochondrial activity using MTT.

### 2.10. Statistical Analysis

All statistical analysis was performed using GraphPad Prism (version 9.3.1). Tissues from the frontal part of cerebral cortex and hippocampus harvested from the embryonic foetuses of one individual Sprague Dawley rat was considered as one culture (*n* = 1). The raw data of the MTT and LDH assays were analysed using two-way ANOVA with treatment and culture ID as factors to account for differences between cultures. Given a significant overall treatment effect, the two-way ANOVA was followed by Dunnett’s post hoc test with comparison to control. For comparison between negative control and control, an unpaired t-test was used. The data from the dose response study were converted to percentage and analysed using nonlinear regression. All data are expressed as means ±standard error of the mean (SEM), and statistical significance was defined as *p*-value < 0.05.

## 3. Results

### 3.1. Immunocytochemistry

#### 3.1.1. IRAP Expression

**Figure 2:**
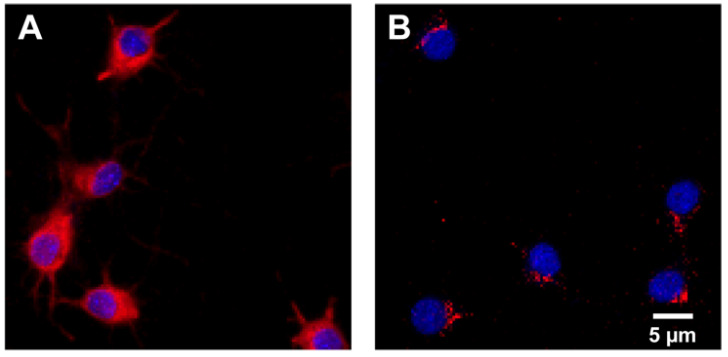
IRAP expression. Representative images of the expression of insulin-regulated aminopeptidase (IRAP) shown in red and cell nuclei stained with 4′,6-diamidino-2-phenylindole (DAPI) shown in blue in (**A**) untreated primary hippocampal and (**B**) cortical cells. IRAP was targeted using primary antibody rabbit anti-IRAP and secondary antibody Alexa 568 anti-rabbit. The images were acquired using ImageXpress (Molecular Devices) mounted with a 20× objective.

The IRAP receptor was expressed in both primary hippocampal and cortical cell cultures. See Figure 2 for representative images of IRAP expression combined with cell nuclei staining using DAPI in hippocampal and cortical cultures.

#### 3.1.2. Distribution of Cell Types

**Figure 3:**
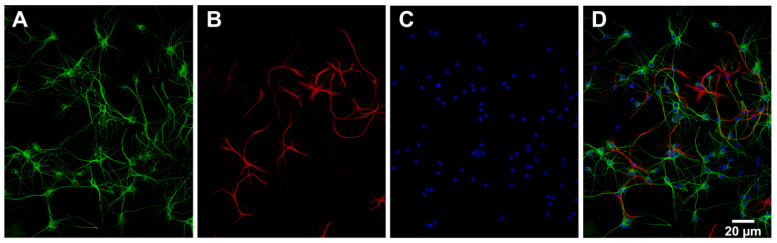
Characterisation of primary hippocampal cell culture. Representative images of (**A**) the expression of microtubule-associated protein 2 (MAP2) shown in green, (**B**) the expression of glial fibrillary acidic protein (GFAP) shown in red and (**C**) the cell nuclei stained with 4′,6-diamidino-2-phenylindole (DAPI) shown in blue in a primary hippocampal culture. (**D**) MAP2 (green), GFAP (red) and DAPI (blue) are merged. The images were acquired using ImageXpress (Molecular Devices) mounted with a 20× objective.

The number of neurons (MAP2 positive cells) and astrocytes (GFAP positive cells) in the primary cell cultures was estimated by analysing the acquired images with an automated ImageJ macro. The analysis was based on images from multiple wells (9 sites per well) of a 96-well plate from one culture of hippocampal and cortical cells. The results showed that the distribution of cell types in hippocampal cultures was 78% neurons, 12% astrocytes and 10% other (most likely other glial cells such as oligodendrocytes and microglia). The distribution of cell types in cortical cultures was 73% neurons, 1% astrocytes and 26% other. Figure 3 shows example images of MAP2, GFAP and DAPI that were used to estimate the cell type distribution in a hippocampal culture.

### 3.2. Dose Response Study

#### Determination of LD_50_

**Figure 4:**
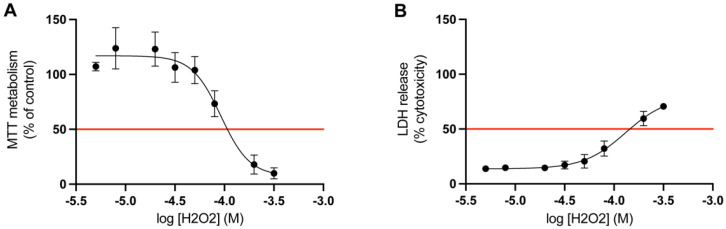
Determination of LD_50_ for hydrogen peroxide. The results of the dose response study on primary cortical cells. The red line marks the LD_50_ value of hydrogen peroxide (H_2_O_2_) for (**A**) MTT metabolism and (**B**) LDH release, which was 0.9 × 10^−4^ M and 1.3 × 10^−4^ M, respectively (*n* = 3). All data are presented as means ± SEM.

The appropriate concentration of hydrogen peroxide was determined by a dose-response study. The LD_50_ value for mitochondrial activity and LDH release was calculated for cortical cells treated with hydrogen peroxide for 24 h (*n* = 3). The LD_50_ value for the MTT metabolism (mitochondrial activity) was 0.9 × 10^−4^ M, and for the LDH release (membrane integrity) it was 1.3 × 10^−4^ M (Figure 4). Given the results acquired from these studies, the optimal concentration of hydrogen peroxide to induce LD_50_ in both of these assays was determined to be 1 × 10^−4^ M. Therefore, in the following assays the concentration of hydrogen peroxide was set to 1 × 10^−4^ M.

### 3.3. Mitochondrial Activity

#### The Effect of HA08 on Mitochondrial Activity

**Figure 5:**
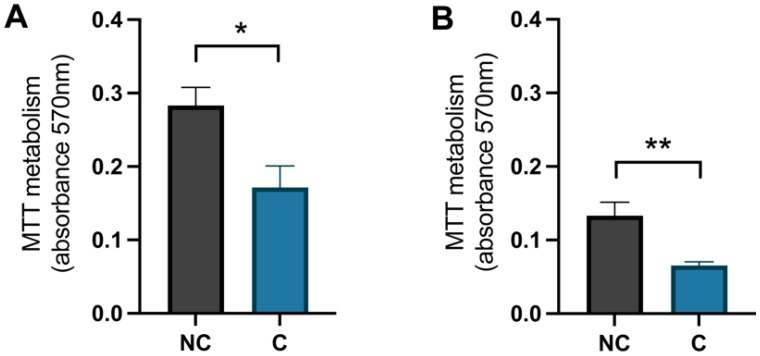
The effect of hydrogen peroxide on mitochondrial activity. The levels of metabolised MTT for (**A**) hippocampal cells and (**B**) cortical cells, negative control (NC) cells compared to positive control (C) cells. NC was treated with MQH_2_O from days 8–10 and C with hydrogen peroxide from days 8–9 and 0.1% DMSO from days 9–10. There was a significant decrease in mitochondrial activity in the control cells compared to the negative control cells in both hippocampal and cortical cultures (*n* = 5). All data are presented as means ± SEM, * *p*-value < 0.05, ** *p*-value < 0.01.

To confirm that the cells were damaged by the hydrogen peroxide, a negative control (NC) was used and these cells were treated with Milli-Q^®^ water (MQH_2_O) from day 8–10. The positive control cells (C) were treated with 1 × 10^−4^ M hydrogen peroxide from days 8–9 and with 0.1% dimethyl sulfoxide (DMSO) from days 9–10. The analysis of the MTT metabolism, which represents the activity of the mitochondria, showed that there was a significant difference between the negative control cells and the positive control cells for both hippocampal and cortical cells (*p*-value 0.0195 and 0.0073, respectively) (Figure 5). The negative control cells had a higher value, meaning the mitochondrial activity was higher in these cells compared to the positive control cells that were treated with hydrogen peroxide.

**Figure 6:**
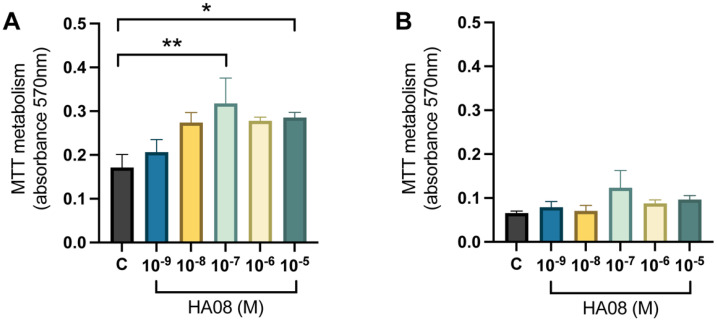
The effect of HA08 on mitochondrial activity. The results of the mitochondrial activity for (**A**) hippocampal cells and (**B**) cortical cells after HA08 treatment. The control cells (C) were treated with 1 × 10^−4^ M hydrogen peroxide from days 8–9 and 0.1% DMSO from days 9–10. The remaining treatment groups (HA08 10^−9^, 10^−8^, 10^−7^, 10^−6^, 10^−5^ M) were treated with 1 × 10^−4^ M hydrogen peroxide from days 8–9 and HA08 of respective concentration days 9–10. There was a significant increase in mitochondrial activity at HA08 concentrations 10^−7^ and 10^−5^ M compared to control cells in hippocampal cultures (*n* = 5). The cortical cells showed no overall effect of treatment (*n* = 5). All data are presented as means ± SEM, * *p*-value < 0.05, ** *p*-value < 0.01.

After the induced cell toxicity with hydrogen peroxide from days 8–9, the cells were treated with different concentrations of HA08 dissolved in 0.1% DMSO from days 9–10. The control cells were treated with 0.1% DMSO. The analysis of the mitochondrial activity (measured as MTT metabolism) for the primary hippocampal cell cultures showed that there was an overall treatment effect between the different groups (*p*-value 0.0155). Further post hoc analysis showed that cells treated with 1 × 10^−7^ and 1 × 10^−5^ M HA08 had a significantly higher mitochondrial activity in comparison to the control cells (*p*-value 0.0072 and 0.0411, respectively); see Figure 6A. The numerical difference of the group means when compared to the control group (level of MTT metabolism 0.1714) was +20%, +60%, +85%, +62% and +67%, respectively for concentrations 1 × 10^−9^, 1 × 10^−8^, 1 × 10^−7^, 1 × 10^−6^ and 1 × 10^−5^ M. For the primary cortical cell cultures, there was no overall effect of treatment (*p*-value 0.1707) and no further post hoc analysis was performed (Figure 6B). The numerical difference of the group means when compared to the control group (level of MTT metabolism 0.0656) was +20%, +8%, +88%, +34% and +47%, respectively, for concentrations 1 × 10^−9^, 1 × 10^−8^, 1 × 10^−7^, 1 × 10^−6^ and 1 × 10^−5^ M.

### 3.4. LDH Release

#### The Effect of HA08 on Membrane Integrity

**Figure 7:**
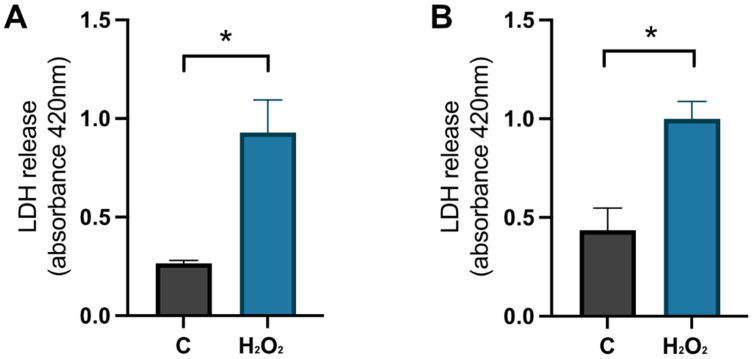
The effect of hydrogen peroxide on membrane integrity. The results of LDH release on day 9 for control cells (C) treated with MQH_2_O and hydrogen-peroxide treated cells (H_2_O_2_). There was a significant increase in LDH release for the cells treated with hydrogen peroxide when compared to control cells in (**A**) hippocampal cultures (*n* = 3) and (**B**) cortical cultures (*n* = 3). All data are presented as means ± SEM, * *p*-value < 0.05.

To confirm that the cells were damaged by the hydrogen peroxide, a negative control (NC) was used. These cells were treated with MQH_2_O from days 8–10. The positive control cells (C) were treated with 1 × 10^−4^ M hydrogen peroxide from days 8–9 and with 0.1% DMSO from days 9–10. The analysis of the LDH release in the cell media, which represents the level of membrane integrity, showed that there was a significant increase of LDH in the positive control compared to the negative control for primary hippocampal (*p*-value 0.0159) and cortical (*p*-value 0.0167) cultures on day 9 (see Figure 7). This confirms that the cells were damaged by the hydrogen peroxide treatment before initiating the HA08 treatment.

**Figure 8:**
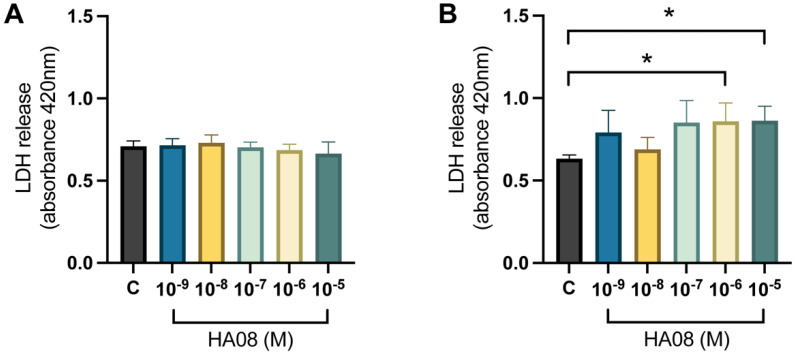
The effect of HA08 on membrane integrity. The results of the LDH release for (**A**) hippocampal cells and (**B**) cortical cells after HA08 treatment. The control cells (C) were treated with 1 × 10^−4^ M hydrogen peroxide from days 8–9 and 0.1% DMSO from days 9–10. The remaining treatment groups (HA08 10^−9^, 10^−8^, 10^−7^, 10^−6^, 10^−5^ M) were treated with 1 × 10^−4^ M hydrogen peroxide from days 8–9 and HA08 of respective concentration from days 9–10. The hippocampal cells showed no significant difference in LDH release at any HA08 concentration compared to control cells (*n* = 5). The cortical cells showed a significant increase in LDH release at HA08 concentrations 10^−6^ and 10^−5^ M when compared to control cells (*n* = 5). All data are presented as means ± SEM, * *p*-value < 0.05.

After the induced cell toxicity with hydrogen peroxide, the cells were treated with different concentrations of HA08 dissolved in 0.1% DMSO from days 9–10. The control cells were treated with 0.1% DMSO. The analysis of the membrane integrity (measured as LDH release) for the primary hippocampal cell cultures showed no overall treatment effect between the different groups (*p*-value 0.3971); see Figure 8A. The numerical difference of the group means when compared to the control group (level of LDH release 0.709) was +1%, +3%, −1%, −3% and −6% for concentrations 1 × 10^−9^, 1 × 10^−8^, 1 × 10^−7^, 1 × 10^−6^ and 1 × 10^−5^ M. The results for the primary cortical cell cultures showed that there was an overall treatment effect between the different groups (*p*-value 0.0292). Further post hoc analysis showed that cells treated with 1 × 10^−6^ and 1 × 10^−5^ M HA08 had an increase in LDH release in comparison to the control cells (*p*-value 0.0251 and 0.0221, respectively); see Figure 8B. The numerical difference of the group means when compared to the control group (level of LDH release 0.6328) was +25%, +9%, +35%, +36% and +36% for concentrations 1 × 10^−9^, 1 × 10^−8^, 1 × 10^−7^, 1 × 10^−6^ and 1 × 10^−5^ M.

## 4. Discussion

This study was performed using mixed glial and neuronal primary cortical and hippocampal cell cultures to mimic a more physiological relevant cell culture condition in comparison to established cell lines. The overall results in the present study demonstrate that HA08 has a restorative effect on mitochondrial activity in rat primary hippocampal cell cultures after being exposed to hydrogen peroxide. The mitochondrial activity in the primary cortical cell cultures was, however, not affected by the HA08 treatment. The cell cultures used for both the hippocampal and cortical studies were intact and expressed IRAP as demonstrated by immunocytochemistry. The main cell type present in the primary cultures was estimated to be neurons (70–80%), indicating that the observed effects were primarily neuronal effects. The ability of HA08 to facilitate restorative effects in neurons gives further strength to its potential cognitive role and the ability to recover cognitive impairment [2,12,25,26,29,30,34].

The assays used in this study measure different types of cell viability. In the MTT assay, it is the mitochondrial response (metabolic activity) that is measured [41,42], meaning that decreased viability here indicates cell death most likely in the form of apoptosis [43]. In the LDH assay, it is rather an uncontrolled cell death that is measured. Decreased viability in the form of increased LDH levels in the cell media indicates a high level of membrane damage [44], which is a typical necrotic response [43]. The results of the MTT assay provide data on the amount of MTT that has been metabolised. However, the assay does not distinguish if this is a change in individual mitochondrial function or in the number of mitochondria in the cells. It may also reflect an effect on the number of cells. Overall, it can be interpreted as cell viability, although the exact mechanism behind the results is unclear.

The results of the mitochondrial activity showed a significant increase at HA08 concentrations 0.1 μM and 10 μM in primary hippocampal cell cultures when compared to control. Furthermore, the *p*-value of the post hoc test when comparing between control and HA08 1 μM was 0.0606, and the numerical differences between the means of 1 μM and 10 μM were negligible. The overall trend of the mitochondrial activity for hippocampal cell cultures can therefore be compared to a bell-shaped dose-response curve; it seems to peak at 0.1 μM and then decrease at higher concentrations (Figure 6A). A bell-shaped dose-response effect of HA08 is also supported by a previous study [30], in which Diwakarla et al. 2016 reported that HA08 increased the number of dendritic spines in primary hippocampal cell cultures at 0.1 μM and 1 μM, but not at higher concentrations [30]. In this latter case, the HA08 treatment was repeated, in comparison to this study, in which we applied an acute treatment regime. The primary cortical cell cultures were, however, unaffected in mitochondrial activity by the treatment. Although not significant, there was a large numerical increase in the mean of HA08 at 0.1 μM when compared to the control group. Overall, the level of mitochondrial activity for the cortical cells was approximately 2 to 3 times lower when comparing the control groups of cortical and hippocampal cultures (cortical negative control 47% lower and positive control 62% lower compared to hippocampal cells). Thus, it is tempting to speculate that the primary cortical cells are more sensitive to the exposure of hydrogen peroxide or perhaps there is an overall lower mitochondrial activity in this cell type.

The results of the membrane integrity measurement showed no overall treatment effect in primary hippocampal cell cultures. Thus, the membrane integrity of the cells remains stable both before and after HA08 treatment. In this aspect, HA08 can be concluded to be non-toxic, since no induced cell death was observed in primary hippocampal cells. On the contrary, the results for the primary cortical cell cultures showed increased LDH levels with increased concentration of HA08 in this cell type. In general, HA08 tends to increase the LDH release, especially at the higher concentrations. This implies that the cortical cells might be more sensitive to membrane damage when compared to the hippocampal cells and that HA08 has a negative impact on the cells under these conditions. Taken together with the previously discussed mitochondrial activity, it can be concluded that the cortical cells are in general more sensitive compared to the hippocampal cells. To ensure that both the cortical and hippocampal cell cultures expressed IRAP, immunocytochemistry was performed using antibodies targeting IRAP. Surprisingly, a higher density of the IRAP receptor was visually observed in the hippocampal cell cultures that further explains the effects seen in hippocampal cells. This warrants further studies examining the differences in IRAP expression in both hippocampal and cortical cell cultures as this study was not designed for this purpose. Nevertheless, the role of IRAP might be less prominent in the cortex compared to the hippocampus, which is the major region for creating and storing memory [45,46]. Furthermore, it is tempting to speculate whether IRAP has different roles in different brain regions [3]. The hippocampus is known to be the main region involved in cognitive functions such as memory and learning [45,46], whereas the prefrontal cortex (PFC) is known to be a process center in the brain, i.e., where stimuli are processed in different ways but not stored or retrieved. In the PFC, relevant information is selected, processed and controlled; thus, PFC is suggested to be responsible for cognitive control [47,48].

As discussed above, the distribution of IRAP may differ between brain regions and cell types. The outcome of higher or lower levels of IRAP activity may affect the concentration of substrates, the balance and the number of products formed after the processing of IRAP. IRAP deficiency has, for example, been shown to have protective effects in a mice model of ischemic stroke in which IRAP knockout mice had less neurological impairment and smaller infarct volumes compared to wild-type mice [39]. The endogenous IRAP inhibitor Ang IV has also been shown to have protective effects against induced ischemia, in which Ang IV-treated rats showed less neurological deficit, reduced infarct and overall reduced mortality [40]. In addition, Ang IV has been studied directly in relation to its effect on memory, where it has been shown to improve or restore memory in different animal models [2,16,17,18,19,37,38], including long-term treatment of an Alzheimer’s disease mice model [37]. It has also been shown to increase the amount of brain-derived neurotrophic factor (BDNF) and decrease GABA, the main inhibitory neurotransmitter [38].

The present study was designed as an explorative study and as such, no mechanistical data are provided. The mechanism by which HA08 exerts its effects remains unclear and further studies are needed to reveal the mechanism behind both the protective and restorative effects.

To conclude, inhibitors of IRAP, such as Ang IV, improve memory and cognition in animal models, and the aminopeptidase IRAP is recognized as a new potential target for drugs aimed at treatment of cognitive disorders [2,9,16,17,18,19,37,38]. As a consequence, in recent years a large number of IRAP inhibitors have been reported from different laboratories [9,22,25,26,27,28,29,30,31,32] and HA08, a macrocyclic disulfide analogue of the unstable endogenous Ang IV, has been identified as one of the most potent IRAP inhibitors known to date [12,34]. HA08 enhances dendritic spine density in rat hippocampal primary cultures [30], and as reported herein, restores cell viability by increasing the mitochondrial activity in primary hippocampal cultures after hydrogen-peroxide-induced damage. The observation that the viability of the damaged cells could be restored by blocking IRAP with HA08, combined with the previously reported data on this compound and related IRAP inhibitors, has now encouraged a search for and design of new IRAP inhibitors, with favourable pharmacokinetic profiles that also are more metabolically stable than HA08. Such IRAP inhibitors should be able to cross the blood brain barrier (BBB), and address IRAP in the brain to restore functions that have been compromised by disease or trauma.

## Data Availability

Not applicable.

## Abbreviations

Ang IV	Angiotensin IV
ANOVA	Analysis of variance
DAPI	4′,6-diamidino-2-phenylindole
DIV	Days in vitro
DMSO	Dimethyl sulfoxide
GFAP	Glial fibrillary acidic protein
H_2_O_2_	Hydrogen peroxide
IRAP	Insulin-regulated aminopeptidase
LDH	Lactate dehydrogenase
MAP2	Microtubule-associated protein 2
MQH_2_O	Milli-Q^®^ water
MTT	Tetrazolium bromide salt
NBM	Neurobasal plus media
SEM	Standard error of the mean
